# Citrate-Buffered, Magnesium-Enriched Dialysate on Calcification Propensity in Hemodialysis Patients – The CitMag Study

**DOI:** 10.1016/j.ekir.2024.03.023

**Published:** 2024-03-25

**Authors:** Daniel Cejka, Ursula Thiem, Eric Blinzler, Jennifer Machacek, Jakob Voelkl, Edward R. Smith, Andreas Pasch, Maria C. Haller

**Affiliations:** 1 Department of Medicine III – Nephrology, Hypertension, Transplantation, Rheumatology, Geriatrics, Ordensklinikum Linz - Elisabethinen Hospital, Linz, Austria; 2 Faculty of Medicine, Johannes Kepler University Linz, Austria; 3 Institute for Physiology and Pathophysiology, Johannes Kepler University Linz, Austria; 4 Department of Nephrology and Medical Intensive Care, Charité-Universitätsmedizin Berlin, Berlin, Germany; 5 DZHK (German Centre for Cardiovascular Research), Partner Site Berlin, Berlin, Germany; 6 Department of Nephrology, Royal Melbourne Hospital, Parkville, Victoria, Australia; 7 Department of Medicine, University of Melbourne, Parkville, Victoria, Australia; 8 Calciscon AG, Biel, Switzerland; 9 Center for Medical Statistics, Informatics, and Intelligent Systems, Medical University, Vienna, Austria

**Keywords:** calcification propensity, citrate, dialysis, magnesiumT50

## Abstract

**Introduction:**

Serum calcification propensity (T50 time) is associated with mortality in patients on dialysis. Several solitary interventions improve T50. However, whether a combination of interventions yields further increases in T50 is unknown. We hypothesized that a combination of 2 interventions, namely increasing magnesium concentration while simultaneously substituting acetate for citrate in the dialysis fluid, leads to increases in T50 values.

**Methods:**

In a randomized controlled trial, 60 patients on chronic hemodialysis were allocated to either continue on standard (S) dialysate (3 mmol/l acetate, 0.5 mmol/l magnesium) or a sequence of magnesium-enriched (Mg_0.75_) dialysate (3 mmol/l acetate, 0.75 mmol/l magnesium) for 2 weeks followed by combination treatment using citrate-buffered, magnesium-enriched (Cit+Mg_0.75_) dialysate (1 mmol/l citrate, 0.75 mmol/l magnesium) for 3 weeks. The primary end point was the difference in T50 times between the S group and the Cit+Mg_0.75_ group.

**Results:**

There was no significant difference in T50 time between the S group and the Cit+Mg_0.75_ group (236 ± 77 vs. 265 ± 97 min, *P* = 0.23). The size (hydrodynamic radius) of secondary calciprotein particles did not differ between the S group and the Cit+Mg_0.75_ group (294 ± 95 vs. 309 ± 91 nm, *P* = 0.56). In longitudinal analyses, serum magnesium concentrations increased from 1.07 ± 0.17 to 1.24 ± 0.17 mmol/l with the Mg_0.75_ dialysate (*P* < 0.0001) but decreased again to 1.19 ± 0.16 mmol/l with the Cit+Mg_0.75_ dialysate (*P* < 0.0001).

**Conclusion:**

The combination of citrate buffer with increased magnesium concentration in dialysate does not improve T50.

## Graphical abstract

**Figure:**
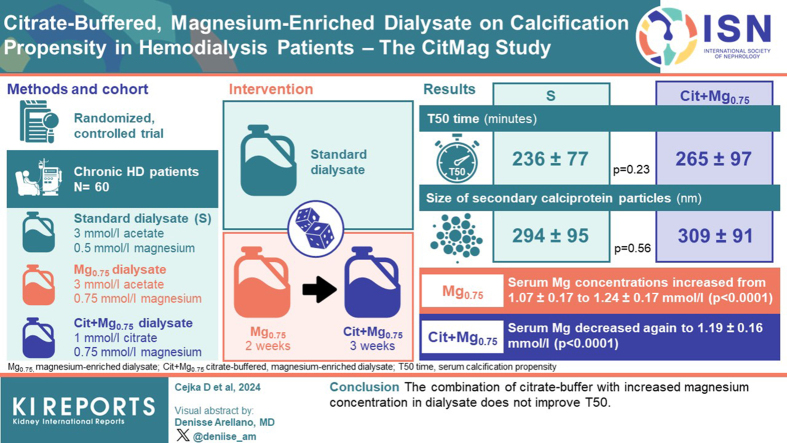

People with chronic kidney disease (CKD) and especially patients on dialysis are at very high risk for cardiovascular events and death.^1^^,^^2^ The exceptionally high burden of cardiovascular disease is substantially mediated by vascular damage induced by abnormalities of mineral and bone homeostasis, which occur regularly in patients with CKD and are summarized as CKD-mineral and bone disorder syndrome.^3^

Measurement of T50 time integrates the effect of several promoters (e.g., calcium and phosphate) and inhibitors (e.g., fetuin-A, magnesium, and bicarbonate) of vascular calcification into a single readout.^4^ Low T50 times have been associated with an increased risk of cardiovascular morbidity and mortality in a broad spectrum of individuals, including the general population,^5^ patients with ischemic heart failure,^6^ patients with peripheral arterial disease,^7^ patients with CKD,^8^^,^^9^ kidney transplant recipients,^10^^,^^11^ and patients on dialysis.^12^, ^13^, ^14^ Therefore, interventions to improve calcification propensity (increase T50 times) are of great interest to potentially improve patient outcomes. Several interventions have been reported to increase T50 times in patients on dialysis, including dialysis treatment *per se*,^15^^,^^16^ phosphate-binder therapy,^17^^,^^18^ use of citrate-buffered instead of acetate-buffered dialysate,^19^^,^^20^ and increasing magnesium concentration in dialysate.^21^ Combinations of different interventions to maximize T50 appear theoretically attractive but have not been studied so far. Therefore, the aim of the study was to investigate the effect of a combination of substituting acetate for citrate and increasing magnesium concentration in dialysate on calcification propensity (T50) in patients on hemodialysis.

## Methods

### Patients

The patients were recruited at the dialysis facility of the Ordensklinikum Linz - Elisabethinen, which is a tertiary care nephrological center. The main inclusion criteria were age ≥ 18 years and chronic (≥ 3 months) dialysis treatment with thrice weekly hemodialysis or hemodiafiltration. The main exclusion criteria were history of intolerance of citrate dialysate, parathyroidectomy planned or expected, scheduled living donor kidney transplant, therapy with bisphosphonates within the past 12 months, and treatment with denosumab within the past 6 months. The first patient was recruited on November 11, 2021. The last patient visit was on December 15, 2022.

### Intervention

This was a prospective, randomized, 1:1 allocation, parallel-group, open-label, single-center study.

A randomization list was generated by an independent statistician using permuted blocks randomization in SAS software (SAS Institute, Cary, NC). The independent statistician prepared opaque, sealed, and consecutively numbered envelopes containing the respective sequence allocation. Patients were assigned in turn to the next consecutive number. The independent statistician had no further participation in the study.

Patients who were randomized to the control arm continued treatment with centrally prepared S dialysate (Granumix plus, Fresenius Medical Care [FMC], Bad Homburg, Germany) containing 3 mmol/l acetate, 0.5 mmol/l magnesium, and 1.25 mmol/l calcium throughout the study (S group). Patients randomized to the intervention arm were sequentially treated with a magnesium-enriched acetate-buffered dialysate first (Diamix AC-F canisters, FMC) containing 3 mmol/l acetate, 0.75 mmol/l magnesium, and 1.25 mmol/l calcium (Mg_0.75_ group), which was followed by treatment with magnesium-enriched, citrate-buffered dialysate (Smartbag CA, FMC) containing 0 mmol/l acetate, 1 mmol/l citrate, 0.75 mmol/l magnesium, and 1.5 mmol/l calcium (Cit+Mg_0.75_ group). A higher calcium-concentration in the citrate-buffered dialysate is needed to compensate for complexation of calcium with citrate in order to maintain stable serum calcium levels.^20^ As opposed to citrate-dialysis which is used for extracorporal anticoagulation during dialysis treatment, additional infusion of calcium is not necessary due to the increased calcium content of citrate-buffered dialysate. Finally, patients in the intervention group were switched back to S dialysate during the 3-week wash-out phase to study the potential reversibility of changes in T50 and other parameters. Bicarbonate was added to all dialysates by each dialysis machine (model 6008, FMC) during the dialysis treatment using dry bicarbonate concentrate (Bibag, FMC), to yield a dialysate-bicarbonate concentration of 35 mmol/l throughout the study. Enoxaparin at a standard dose of 4.000 IU (dose modified as clinically needed) was administered at the beginning of every dialysis session as intradialytic anticoagulant. Dialysis dose (Kt/V) was monitored at every dialysis session using an online clearance monitoring system (OCM and FMC) and remained stable throughout the study. Because of concerns regarding an increased incidence of intradialytic muscle cramps with citrate-buffered dialysate containing 0.5 mmol/l magnesium as reported previously,^22^ a study arm using citrate-buffered dialysate with 0.5 mmol/l magnesium was omitted from the study design.

Dosing of oral phosphate binders; calcimimetics; vitamin D preparations; and concentrations of magnesium, calcium, and bicarbonate p.o. or in the dialysis bath were kept constant as far as clinically justifiable, in order to avoid interference with calcification propensity. However, physicians in charge of routine patient care were free to change any medication or dialysis prescription during the study if deemed clinically necessary.

In total, 8 study-specific visits per patient were scheduled. Blood was drawn immediately before initiation of hemodialysis via the dialysis access (arteriovenous fistula or central venous catheter). In patients who had a central venous catheter as dialysis access, 10 ml of blood was drawn and discarded before blood sampling for study purposes. In the run-in-phase which lasted 1 week, visits were scheduled before the first and second dialysis session of the week. All other study phases in the intervention group were scheduled to last 3 weeks, and study visits were scheduled before the first and second dialysis session of the third week. Because of dialysate supply chain interruptions due to the COVID-19 pandemic during the ongoing study, the study phase using magnesium-enriched, acetate-buffered dialysate (Mg_0.75_) had to be shortened from 3 to 2 weeks.

### Laboratory Measurements

Blood was collected in gel separator tubes (SST, BD Vacutainer, Becton Dickinson, Plymouth, UK) for serum and K_3_EDTA tubes (Vacuette, Greiner BioOne, Kremsmünster, Austria) for plasma. Samples were centrifuged at 1280 rcf for 15 minutes at room temperature and aliquots were transferred directly to −80 °C for long-term storage. Calcification propensity was measured in serum using the T50 test as described previously^12^ at Calciscon (Calciscon AG, Biel, Switzerland) without knowledge of the randomization group or study phase. The hydrodynamic radius of secondary calciprotein particles generated in the T50 test at 600 minutes, which has been associated with mortality in patients on dialysis,^23^ was measured using 3D-dynamic light scattering as described previously^12^^,^^24^ at Calciscon. Routine blood tests were performed in the central laboratory facility of the Ordensklinikum Linz - Elisabethinen hospital using Cobas analyzer systems (Roche Diagnostics, Rotkreuz, Switzerland). Blood gas analysis was performed using the RAPIDpoint 500 point-of-care blood gas system (Siemens Healthcare Diagnostics, Vienna, Austria). Intact parathyroid hormone was measured using the Elecsys PTH (1-84) assay on a Cobas system (Roche).

### Sample Size Calculation and Statistical Methods

#### Sample Size Calculation

On the basis of previously published data on the effect of increasing magnesium in dialysate^21^ and substituting acetate for citrate^19^^,^^20^ as dialysis buffer, increases in T50 of 25 minutes per 0.25 mmol increase in dialysate magnesium and 30 minutes by switching from 3 mmol/l acetate buffer to 1 mmol/l citrate buffer were expected. The sample size needed was calculated using nQuery Advisor software (Statistical Solutions, Cork, Ireland) was 40 patients (20 per group), assuming a mean T50 value at baseline of 220 minutes, a total increase in T50 in the Cit+Mg_0.75_ group of 55 minutes, an SD in T50 of 60 minutes, an α-level of 0.05, a 2-sided test, and a statistical power of 80%. To account for the usually high drop-out rate in patients on dialysis due to high burden of disease (frequent hospitalizations due to acute illness, deaths, or kidney transplantations), the sample size was increased to 60 patients (30 per group).

#### Statistics

Patient characteristics at inclusion were described by mean and SD, median and interquartile range, or frequency and percentage for data with normal distribution, data with skewed distribution, and categorical variables, respectively. The primary hypothesis was that the T50 value in the patient group treated with acetate-free citrate-containing dialysate with increased magnesium (0.75 mmol/l) concentration (Cit+Mg_0.75_ group) would differ from the T50 value of the S group (acetate-based dialysate containing 0.5 mmol/l magnesium) at 5 weeks. Mean values of T50 and other parameters were calculated from both visits (i.e., after a “long” and a “short” interdialytic interval) in each study phase to account for intrapatient variability and increase robustness of results. Statistical analyses were performed using these mean values, unless stated otherwise. The primary analysis was performed per protocol and compared T50 values (showing normal distribution) in patients who completed the Cit+Mg_0.75_ phase as compared to the control group arm using unpaired *t*-test. Prespecified secondary analyses included a comparison of the T50 time between S group and Cit+Mg_0.75_ group using only the midweek dialysis session data to estimate a steady state condition (as opposed to the first dialysis session of the week after a “long” interval) and a comparison of the T50 time between the Mg_0.75_ group and the S group, using *t*-test. Additional prespecified analyses were studies of longitudinal changes in T50 in each study arm and between study phases (linear mixed model) and the influence of T50 and magnesium values at baseline on changes in T50 (linear regression). *P*-values < 0.05 (2-tailed) were considered statistically significant.

### Ethics

This study was conducted according to regulations of the International Conference on Harmonization on Good Clinical Practice and the Declaration of Helsinki on Ethical Principles for Medical Research Involving Human Subjects. Patients were only included in this study after providing oral and written informed consent. This study was reviewed and approved by the Ethics Committee of the Johannes Kepler University Linz (ID: 1086/2019) and prospectively registered in the European Union Drug Regulating Authorities Clinical Trials Database (ID: 2019-001798-10) on April 15, 2019.

## Results

### Patients

Of 126 screened patients, 60 were randomized and 53 were available for analysis of the primary end point. A CONSORT study flow diagram with further details is shown in Supplementary Figure S1). Patient demographics at baseline (*N* = 60) are shown in Table 1.

**Table 1:** Patient demographics of recruited patients (*N* = 60) at baseline

Demographic, clinical and laboratory characteristics	Overall	Control	Intervention
Patients recruited, *n*	60	30	30
Age (yr), mean ± SD	69 ± 17	69 ± 11	69 ± 10
Sex, men/women, *n*/*n* (%/%)	40/20 (66.7/33.3)	20/10 (66.7/33.3)	20/10 (66.7/33.3)
Body mass index (kg/m^2^), mean ± SD	25.6 ± 4.6	25.4 ± 4.2	25.7 ± 4.9
Primary renal disease, *n* (%)			
Diabetes	18 (30)	7 (23)	11 (36)
Hypertensive/vascular	12 (20)	7 (23)	5 (16)
ADPKD	5 (8.3)	2 (6)	3 (10)
Glomerulonephritis	14 (23.3)	9 (30)	5 (16)
Other	11 (18.3)	5 (16)	6 (20)
Dialysis vintage (mo), median (IQR)	32 (22–59)	34 (23–60)	30 (20–59)
Previous kidney transplants, *n* (%)			
None	52 (86.7)	23 (76)	29 (96)
1	6 (10)	5 (16)	1 (3)
2	1 (1.7)	1 (3)	0 (0)
3	1 (1.7)	1 (3)	0 (0)
Vascular access, *n* (%)			
AV fistula	40 (66.7)	20 (66.7)	20 (66.7)
Catheter	20 (33.3)	10 (33.3)	10 (33.3)
Laboratory parameters baseline, mean ± SD			
Calcium, albumin-corrected (mmol/l)	2.17 ± 0.13	2.18 ± 0.11	2.17 ± 0.14
Phosphate (mmol/l)	1.79 ± 0.38	1.76 ± 0.35	1.76 ± 0.42
Bicarbonate (mmol/l)	21.7 ± 2.5	21.8 ± 2.0	21.6 ± 2.7
Magnesium (mmol/l)	1.02 ± 0.17	1.03 ± 0.14	1.09 ± 0.19
iPTH (pg/dl)	233 (189–380)	238 (195–381)	221 (175–391)
Hemoglobin (g/dl)	11.6 ± 1.0	11.6 ± 1.0	11.7 ± 1.1
Comorbidities, *n* (%)			
Diabetes mellitus	25 (58.3)	9 (33)	16 (53)
Coronary artery disease	27 (45)	15 (50)	12 (40)
History of myocardial infarction	6 (10)	3 (10)	3 (10)
History of congestive heart failure	25 (41)	14 (46)	11 (36)
Atrial fibrillation	7 (11.7)	3 (10)	4 (13)
Peripheral occlusive vascular disease	19 (31.7)	7 (23)	12 (40)
History of amputation	10 (16.7)	3 (10)	7 (23)
Cerebrovascular disease	18 (30)	11 (36)	7 (23)
History of stroke or TIA	11 (18.3)	4 (13)	7 (23)
Cigarette smoking	10 (16.7)	7 (23)	3 (10)
Dialysis treatment, *n* (%)			
HD	20 (33.3)	9 (33)	11 (36)
HDF	40 (66.7)	21 (70)	19 (63)
Duration per session (min)	231 ± 19	231 ± 19	231 ± 19
Kt/V	1.13 ± 0.21	1.16 ± 0.18	1.09 ± 0.24

ADPKD, autosomal dominant polycystic kidney disease; AV fistula, arteriovenous fistula; HD, hemodialysis; HDF, hemodiafiltration; iPTH, intact parathyroid hormone; IQR, interquartile range; Kt/V, dialysis dose measured with online clearance monitoring of plasma sodium; SD, standard deviation; TIA, transitory ischemic attack.

### Calcification Propensity

**Figure 1:**
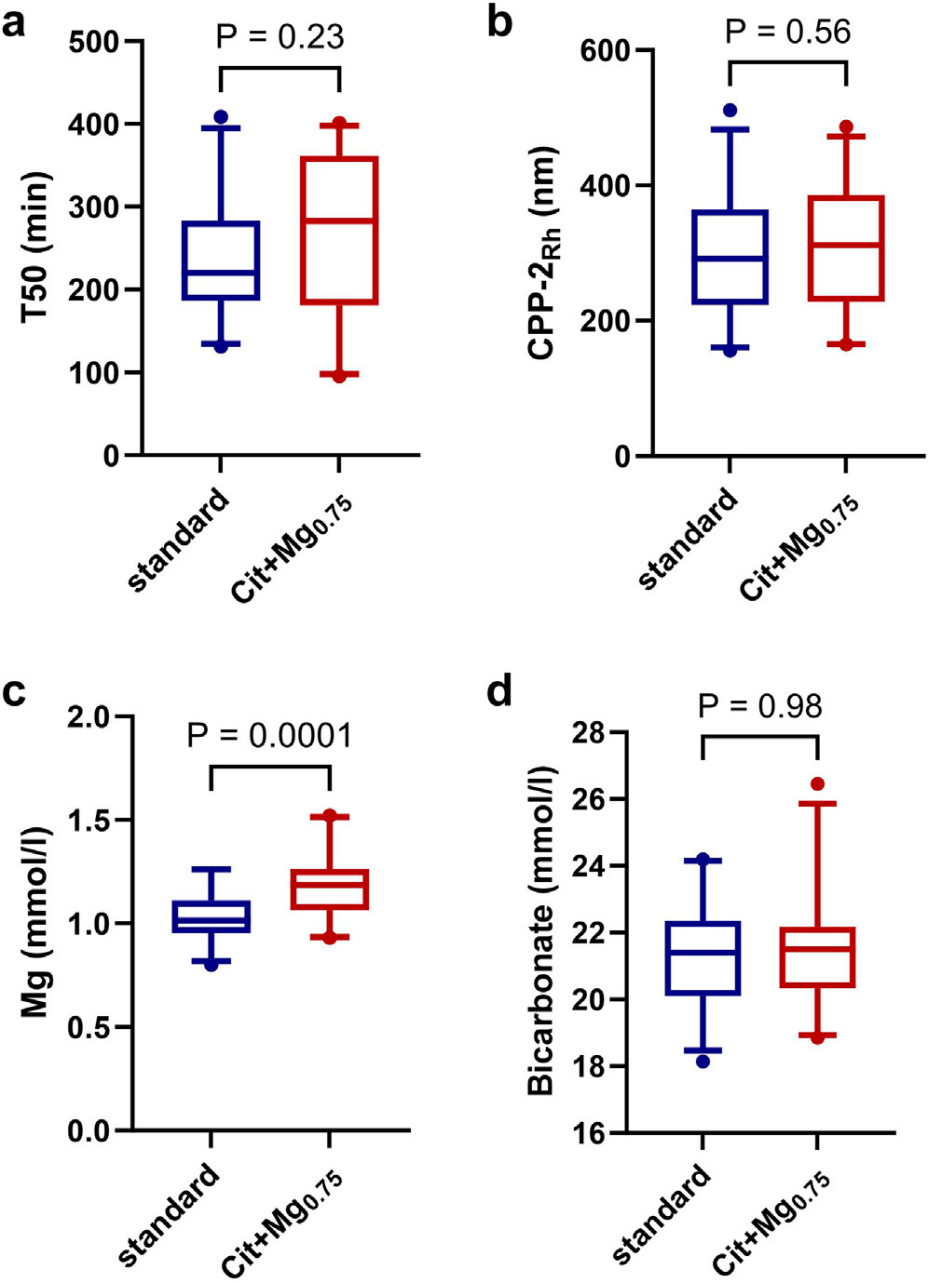
(a) Serum calcification propensity (T50 time), (b) secondary calciprotein size (hydrodynamic radius [Rh]) of secondary calciprotein particles (CPP-2_Rh_), (c) serum magnesium, and (d) serum bicarbonate, in patients treated with standard dialysate (3 mmol/l acetate, 0.5 mmol/l magnesium) compared to citrate-buffered, magnesium-enriched (Cit+Mg_0.75_) dialysate (1 mmol/l citrate, 0.75 mmol/l magnesium).

Regarding the primary end point, there was no significant difference in T50 time between the S dialysate group and the Cit+Mg_0.75_ dialysate group (236 ± 77 vs. 265 ± 97 min, *P* = 0.23; Figure 1a). Using only data from the second dialysis session of the week (midweek session, prespecified secondary end point), T50 values between the S group and the Cit+Mg_0.75_ group did not differ significantly (237 ± 80 vs. 278 ± 95 min, *P* = 0.09). The size of secondary calciprotein particles generated in the T50 test did not differ between the S group and the Cit+Mg_0.75_ group (294 ± 95 vs. 309 ± 91 nm, *P* = 0.56; Figure 1b). Furthermore, the difference in T50 time between the S group and the Mg_0.75_ group (magnesium-enriched, acetate-buffered) did not reach significance (254 ± 85 vs. 298 ± 98 min, *P* = 0.08).

### Serum Magnesium and Bicarbonate

Compared to the S group, serum magnesium levels were significantly higher in the Cit+Mg_0.75_ group (1.02 ± 0.12 vs. 1.19 ± 0.16 mmol/l, *P* < 0.0001; Figure 1c), whereas serum bicarbonate levels did not differ significantly (21.4 ± 1.6 vs. 21.4 ± 1.7 mmol/l, *P* = 0.98; Figure 1d).

### Longitudinal Analyses

**Figure 2:**
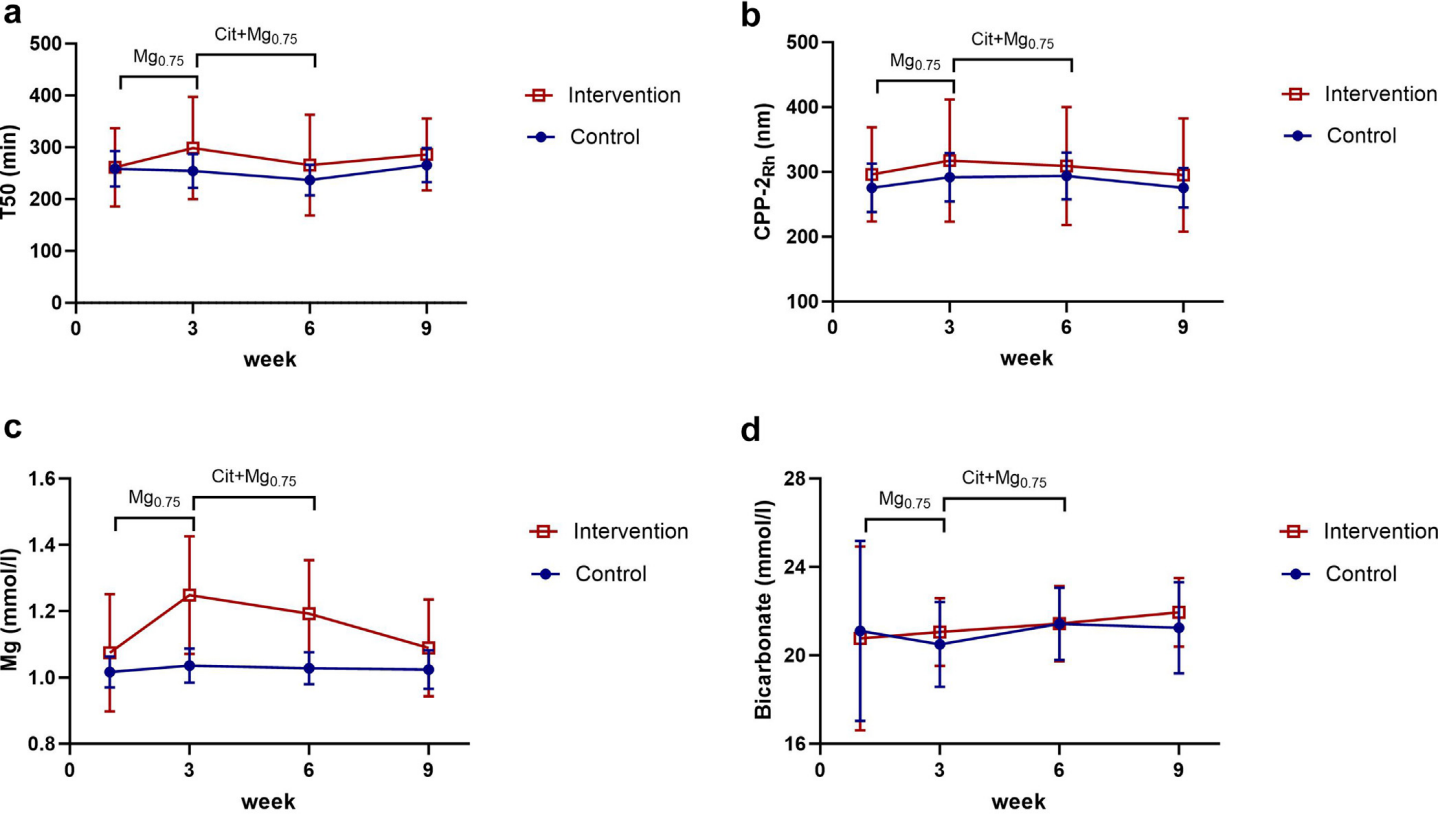
(a) Longitudinal changes (mean, 95% confidence interval) in serum calcification propensity (T50 time, (b) secondary calciprotein size (CPP-2_Rh_), (c) serum magnesium, and (d) serum bicarbonate, in patients treated with standard dialysate (3 mmol/l acetate, 0.5 mmol/l magnesium), magnesium-enriched, acetate-buffered (Mg_0.75_) dialysate (3 mmol/l acetate, 0.75 mmol/l magnesium) and citrate-buffered, magnesium-enriched (Cit+Mg_0.75_) dialysate (1 mmol/l citrate, 0.75 mmol/l magnesium).

In the intervention arm, there was no significant change in T50 (Figure 2a) from baseline with Mg_0.75_ dialysate (261 ± 75 to 298 ± 98 min, *P* = 0.10) or with Cit+Mg_0.75_ dialysate (265 ± 97 min, *P* = 0.55). Similarly, hydrodynamic radius of secondary calciprotein particles (Figure 2b) did not change significantly from a baseline with the Mg_0.75_ dialysate (297 ± 76 to 317 ± 94 nm, *P* = 0.51) or with Cit+Mg_0.75_ dialysate (309 ± 91 nm, *P* = 0.96). In contrast, serum magnesium concentrations (Figure 2c) increased significantly from 1.07 ± 0.17 to 1.24 ± 0.17 mmol/l with the Mg_0.75_ dialysate treatment (*P* < 0.0001), but significantly decreased again to 1.19 ± 0.16 mmol/l in the Cit+Mg_0.75_ dialysate treatment phase (*P* < 0.0001). Serum bicarbonate levels (Figure 2d) did not change significantly from 20.7 ± 4.1 mmol/l at baseline to 21.0 ± 1.5 mmol/l with Mg_0.75_ dialysate (*P* = 1.0) and to 21.4 ± 1.7 mmol/l with Cit+Mg_0.75_ dialysate (*P* = 0.74).

### Exploratory Analyses

Although changes in serum magnesium from baseline (S dialysate) to the Mg_0.75_ dialysate treatment predicted changes in T50 (R^2^ = 0.24, *P* = 0.14), baseline values of neither T50 nor serum magnesium were predictive of the magnitude of changes in T50 per increase in serum magnesium levels (*P* = 0.68 and *P* = 0.62, respectively).

### Other Laboratory Parameters

**Figure 3:**
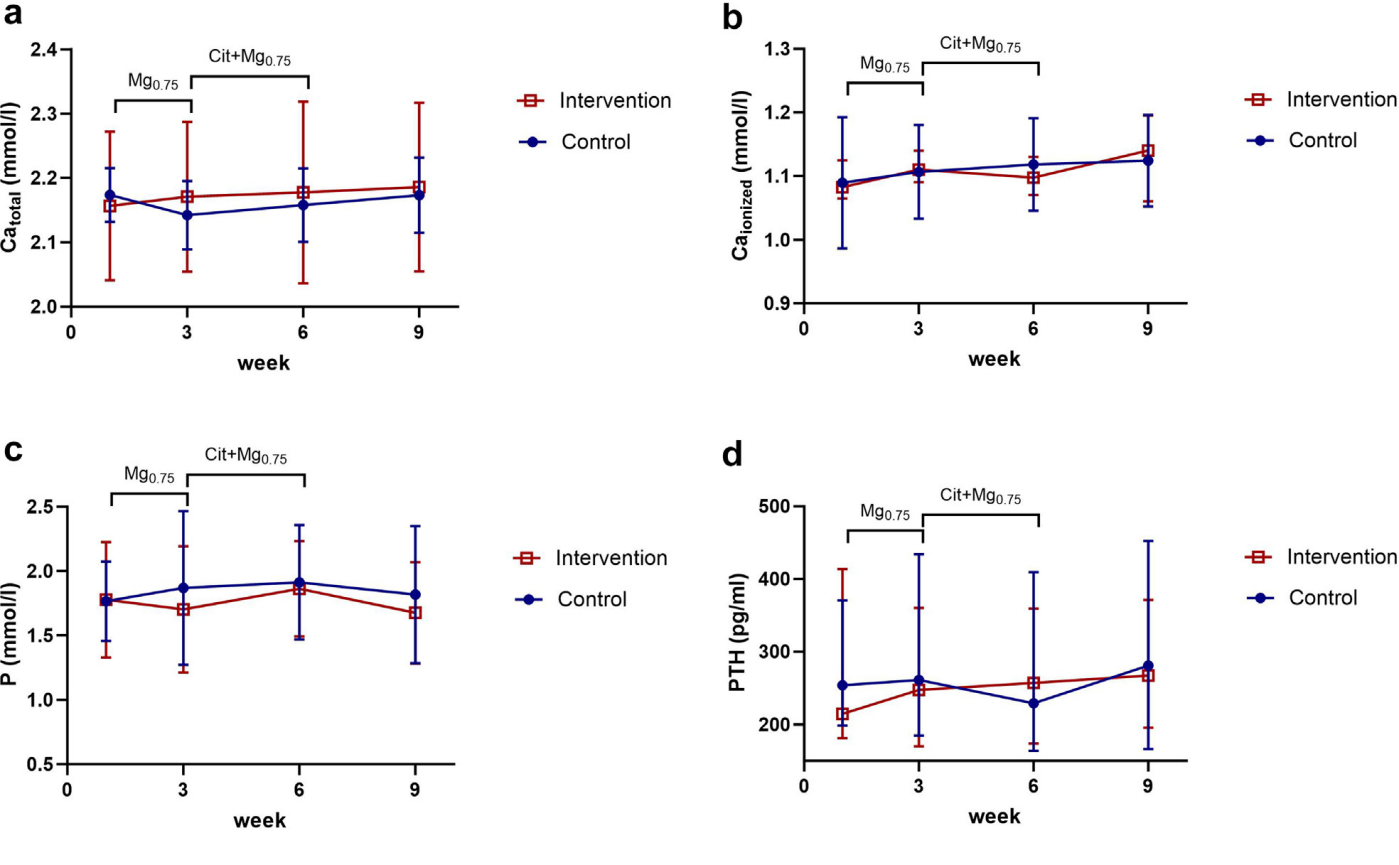
(a) Longitudinal changes (mean, 95% confidence interval) in serum total calcium, (b) serum ionized calcium. (c) serum phosphate, and (d) parathyroid hormone (median and interquartile range), in patients treated with standard dialysate (3 mmol/l acetate, 0.5 mmol/l magnesium), magnesium-enriched, acetate-buffered (Mg_0.75_) dialysate (3 mmol/l acetate, 0.75 mmol/l magnesium) and citrate-buffered, magnesium-enriched (Cit+Mg_0.75_) dialysate (1 mmol/l citrate, 0.75 mmol/l magnesium.

Results of other laboratory measurements, including total calcium, ionized calcium, phosphate, and parathyroid hormone are shown in Figure 3. Taken together, there was no significant difference between the S group and the Cit+Mg_0.75_ group in these parameters (*P* = 0.62, 0.50, 0.67, and 0.36, respectively). In longitudinal analysis, these parameters remained stable over time in both groups. The longitudinal association of biochemical parameters with T50 over the study is summarized in Supplementary Table S1, showing a significant inverse relationship with phosphate and parathyroid hormone levels, and a positive association with magnesium and albumin levels.

### Adverse Events

In total, 59 adverse events and 9 serious adverse events resulting in study drop-outs were observed, as expected in a dialysis population due to high burden of disease. Reasons for study drop-outs according to study arm and study phase are detailed in the CONSORT flowchart in the supplementary material. Reasons for study drop-outs were diverse, including infections, kidney transplantations, and cancer diagnoses. No patient died during the study. None of the drop-outs was deemed to be related to the study dialysate prescriptions, as judged by the local investigators. Generally, dialysis treatment with the Mg_0.75_ as well as the Cit+Mg_0.75_ dialysate was well-tolerated. Of note, the incidence of intradialytic muscle cramps, which has been described to increase with citrate-buffered dialysate,^22^ was low. In total, 5 episodes of intradialytic muscle cramps prompting a change of treatment (e.g., decrease in ultrafiltration rate, administration of 5% glucose i.v., or similar measures) occurred, 1 in the control arm (S dialysate) and 4 in the intervention arm (1 with S dialysate, 2 with Mg_0.75_ dialysate and 1 with Cit+Mg_0.75_ dialysate).

## Discussion

The main finding of our study is that there was no difference in calcification propensity of serum as measured using the T50 test between patients treated with citrate-buffered, magnesium-enriched dialysate as compared to a standard (acetate-buffered) dialysate.

These findings were surprising to some extent. One might speculate that citrate complexed magnesium to a greater extent than acetate, thereby decreasing magnesium serum concentrations. The solubility of magnesium citrate in water at 20 °C (0.44 M) is markedly lower than that of magnesium acetate (1 M).^25^ Indeed, Lorenz *et al.*^19^ reported a subtle but significant decrease in predialytic serum magnesium levels when comparing acetate-buffered with citrate-buffered dialysate, where both dialysate solutions contained 0.5 mmol/l magnesium. However, in the same study the switch from acetate-buffered to citrate-buffered dialysate led to a significant increase in T50 over a period of 3 months, despite decreases in serum magnesium.^19^ Therefore, a switch from acetate-buffered to citrate-buffered dialysate with simultaneous increase in dialysate magnesium concentration to at least offset the citrate-induced decrease in serum magnesium was expected to further increase T50 values, especially given the relatively large effect size of serum magnesium on T50 as reported by Bressendorff and colleagues.^21^ A limitation of the study by Lorenz *et al.*^19^ was its longitudinal design without a simultaneous control group treated with acetate-buffered dialysate. Although T50 increased after the switch from acetate-buffered to citrate-buffered dialysate, this increase in T50 was not reversible upon reswitching back to acetate-buffered dialysate, leaving a possibility for a residual bias in this study.^19^ Indeed, in a randomized controlled cross-over study comparing acetate-buffered with citrate-buffered dialysate over a period of 1 week (again both dialysate solutions containing 0.5 mmol/l magnesium), Ter Meulen *et al.*^20^ did not find significant differences in predialytic T50 times, but only in postdialytic T50 values. This could be explained by the relatively short half-life of citrate in blood of about 50 minutes in patients on dialysis ^26^ because of metabolization to bicarbonate mainly in the liver,^27^ abrogating potential effects of citrate on predialytic T50 values. Again, an increased concentration of magnesium of 0.75 mmol/l in the citrate-buffered dialysate as used in our study would have been expected to still increase T50 values. Nevertheless, in our study, T50 times decreased from Mg_0.75_ dialysate phase to the Cit+Mg_0.75_ dialysate phase to close to baseline values. Although the decrease in T50 times between the Mg_0.75_ phase and the Cit+Mg_0.75_ phase close to baseline values is formally statistically not significant, it still points toward a possible antagonism between citrate and magnesium in dialysate solutions regarding the effect on predialytic T50 time, possibly by complexation and removal of citrate-magnesium complexes by dialysis.

This study has several limitations. The major limitation is the lack of a study arm using a citrate-buffered dialysate with regular (0.5 mmol/l) magnesium concentration. Therefore, the isolated effect of citrate-buffered instead of acetate-buffered dialysate on calcification propensity cannot be assessed in this study. However, a citrate-only study arm was omitted intentionally. In a previous study adverse events, especially intradialytic muscle cramps or pain were reported to be more frequent in a citrate-buffered dialysate containing a regular magnesium concentration of 0.5 mmol/l as compared to an acetate-based dialysate regimen, which may have been mediated to slightly lower post-dialytic serum calcium and magnesium levels in the citrate group compared to the acetate group.^22^ Intradialytic muscle cramps are adverse events which are very poorly tolerated by patients and would probably lead to high discontinuation rates. As magnesium is well known to counteract muscular hyperexcitability and is used for the prevention and treatment of muscle cramps, a treatment phase with an acetate-based dialysate containing an increased (0.75 mmol/l) magnesium dialysate concentration preceding the switch from acetate-buffered to citrate-buffered dialysate seemed prudent. Therefore, a step-up strategy to first increase magnesium in dialysate followed by a switch to a citrate-buffered dialysate while maintaining higher dialysate magnesium concentrations was chosen to minimize potential adverse events and prevent potential muscle cramp-associated drop-outs. Indeed, the rate of muscle cramps was similar and very low in all dialysate groups.

Another limitation is the open-label design of the study. Because the commercially available citrate-buffered dialysate used in this study comes in soft plastic bags, whereas corresponding acetate-buffered dialysate comes in hard plastic canisters, blinding of patients was deemed to be a disproportionate effort hampering feasibility. However, T50 values were measured in the whole batch of samples only after completion of the study, thus eliminating the possibility of T50-guided interventions of patients or physicians alike throughout the study.

A third limitation of the study concerns the timing used regarding duration of the intervention and blood sampling. Postdialytic sampling of patient serum was not performed, thus possible short-lived effects of the citrate-buffered dialysate as seen by Ter Meulen *et al.*^20^ on T50 could not be investigated. Furthermore, blood sampling on dialysis-free days might have revealed effects on T50 which were too short-lived to be detectable after a full interdialytic (2- to 3-day) interval. Whereas increases in dialysate magnesium concentration were reported to prolong T50 within a week,^21^ increases in T50 after switching from acetate-buffered to citrate-buffered dialysate were found after 3 months of treatment.^19^ Therefore, a longer study duration may have yielded different results.

Association between T50 times and patient survival has been consistently reported to be direct and linear. Therefore, increasing T50 times as much as possible holds promise to potentially maximize the clinical benefit. Indeed, the efficacy of single interventions are limited in clinical practice. For instance, lowering phosphate toward normal as recommended by the current Kidney Disease: Improving Global Outcomes guideline for CKD-mineral and bone disorder^3^ often comes at a price of a very high pill burden (sometimes more than 15 phosphate-binder pills/d), which typically and understandably leads to poor patient adherence.^28^ Despite this high pill burden, the phosphate target of near-normal values cannot be achieved in most of patients.^29^ Thus, combinations of several interventions to improve T50 to potentially maximize beneficial effects on patient outcome, do not increase pill burden, and allow for good adherence to therapy such as modifications of dialysate composition, appear attractive. In light of the findings presented here, a combination of citrate buffer with increased magnesium concentration of dialysate does not seem to be a promising approach to maximize predialytic T50. One alternative approach would be to increase dialysate magnesium concentrations further (e.g., 1.0 or 1.25 mmol/l), to counter the putative intradialytic removal of citrate-magnesium complexes. Another alternative approach would be to administer citrate and magnesium sequentially. For instance, the dialysis treatment could be performed using citrate-buffered dialysate, and magnesium could be administered separately at the end of the study by i.v. drip. However, the effects of both approaches on T50 values remain to be studied.

In conclusion, compared to standard dialysate (3 mmol/l acetate, 0.5 mmol/l magnesium), the combination of citrate buffer (1 mmol/l) with increased magnesium concentration (0.75 mmol/l) in dialysate does not improve T50.

## Disclosure

AP is an inventor of the T50 test. He is president of the board of directors, a part-time employee and stockholder of Calciscon AG, Biel, Switzerland, which commercializes the T50 test. ERS is a stockholder and scientific advisor of Calciscon AG. All the other authors declared no competing interests.

## Supplementary Material

Supplementary File (PDF) **Figure S1.** CONSORT study flow diagram. **Table S1.** CONSORT checklist.

## Footnotes

Supplementary File (PDF)
**Figure S1.** CONSORT study flow diagram. **Table S1.** CONSORT checklist.
